# Continuing Cyclin-Dependent Kinase 4/6 Inhibitors Beyond Progression in Advanced Breast Cancer: A Meta-Analysis †

**DOI:** 10.3390/cancers17101609

**Published:** 2025-05-09

**Authors:** Neha Pathak, Sudhir Kumar, Diego Malon Gimenez, Massimo Di Iorio, Jacqueline Savill, Yael Berner-Wygoda, Meredith Li, Consolacion Molto Valiente, Danielle Cuthbert, Aarushi Gupta, Diana P. Arteaga, Atul Batra, Eitan Amir, Abhenil Mittal

**Affiliations:** 1 Division of Medical Oncology and Hematology, Department of Medicine, Princess Margaret Cancer Centre, University of Toronto, 610 University Avenue, Toronto, ON M5G 2C4, Canada; neha.pathak@uhn.ca (N.P.); diego.malongimenez@vitalitenb.ca (D.M.G.); jacqueline.savill@uhn.ca (J.S.); yael.berner-wygoda@uhn.ca (Y.B.-W.); meredith.li@uhn.ca (M.L.); eitan.amir@uhn.ca (E.A.); 2 Division of Medical Oncology and Hematology, Department of Medicine, Odette Cancer Centre, Sunnybrook Health Sciences, University of Toronto, 2075 Bayview Avenue, Toronto, ON M4N 3M5, Canada; sudhir.kumar@sunnybrook.ca; 3 Department of Oncology, Lakeshore General Hospital, 160 Av Still View, Montreal, QC H9R 2Y2, Canada; massimo.di.iorio.med@ssss.gouv.qc.ca; 4 Department of Medical Oncology, Lakeridge Health Centre, Queen’s University, 580 Harwood Avenue, Oshawa, ON L1S 2J4, Canada; cmolto@lh.ca; 5 St Michael’s Hospital, 36 Queen Street East, Division of Medical Oncology and Hematology, Department of Medicine University of Toronto, Toronto, ON M5B 1W8, Canada; danielle.cuthbert@unityhealth.to; 6 Department of Radiology, Health Sciences North, Northern Ontario School of Medicine, 41 Ramsey Lake Road, Sudbury, ON P3E 5J1, Canada; agupta@hsnsudbury.ca; 7 Division of Medical Oncology and Hematology, Department of Medicine, Health Sciences North, Northern Ontario School of Medicine, 41 Ramsey Lake Road, Sudbury, ON P3E 5J1, Canada; parteaga@hsnsudbury.ca; 8 Department of Medical Oncology, Dr. B.R.A. I.R.C.H, All India Institute of Medical Sciences, Aurobindo Marg, New Delhi 110029, Delhi, India; batraatul85@aiims.edu

**Keywords:** cyclin-dependent kinase 4/6 inhibitors, hormone receptor-positive breast cancer, targeted therapies, endocrine resistance

## Abstract

**Background:** The use of cyclin-dependent kinase 4/6 inhibitors (CDK 4/6i) with endocrine therapy (ET) is a first-line standard treatment for hormone receptor-positive (ER+) Human Epidermal Growth factor Receptor-2-negative (HER2-) advanced breast cancer. The data supporting incorporation of CDK 4/6i + ET beyond progression are variable. Here, we report a pooled analysis of this strategy. **Methods:** A systematic review identified reports of both observational and clinical studies, which evaluated the continuation of CDK4/6i beyond progression. The mean overall response rate (ORR) and progression-free survival (PFS) weighted by the study sample size were calculated. Meta-regression comprising linear regression weighted by the sample size (mixed effects) was performed to explore the association between disease and treatment-related factors and the benefit from continuing CDK4/6i. Quantitative significance was assessed using the Burnand criteria. **Results:** Thirteen studies comprising 1530 patients were included. The median age was 58 years, 50.8% had visceral metastases, and 48% had ESR1 mutations; the median lines of prior therapies were 1 (range 1–5), and 96.3% received palbociclib as the initial CDK4/6i. Eight studies tested a CDK4/6i switch as the intervention. The median PFS was 5.3 months, and the ORR was 14%. In randomized studies, statistically significant differences were observed between CDK4/6i continuation and control, although it is uncertain whether the magnitude of the effect is clinically meaningful. Increasing age, lack of prior chemotherapy, no visceral metastasis or ESR1 mutations, and a switch to a non-palbociclib CDK4/6i were associated with better outcomes. **Conclusion:** Continuing a CDK 4/6i + ET beyond progression yields modest benefits. Switching CDK4/6i likely results in improved ORR and PFS. Continuing palbociclib beyond progression is likely ineffectual.

## Simple Summary

Advanced endocrine-driven breast cancer (i.e., cancer which is estrogen/progesterone receptor-positive and HER2-negative) is treated with targeted therapy, consisting of a cyclin-dependent kinase 4/6 inhibitor (CDK 4/6i) and endocrine therapy first, which is a highly effective and well-tolerated treatment. After the cancer stops responding to this strategy, some studies have evaluated continuing the same or switching to a different CDK 4/6i with/without a change in endocrine therapy, with mixed results. We sought to systematically review the evidence and then pool the data in a weighted manner to arrive at a conclusion. We found that this strategy results in modest improvement in outcomes, and certain subgroups (older patients, no chemotherapy, absence of any cancer involvement of the organs, certain mutations like ESR1 mutations, and non-use of palbociclib as the CDK4/6i) did better. This can help to inform clinical decision making and in choosing the right patient for this approach.

## 1. Introduction

Cyclin-dependent 4/6 kinase inhibitors (CDK4/6i) in combination with endocrine therapy have emerged as the standard first-line treatment for advanced (metastatic or unresectable) breast cancers that are estrogen and/or progesterone receptor-positive (ER+) and Human Epidermal Growth factor Receptor-2-negative (HER2-) [1,2,3]. These agents, when used in the first- and second-line setting have demonstrated significant improvements in progression-free survival (PFS) and at least trends for improved overall survival (OS) [4,5,6,7,8]. These oral drugs are well tolerated with hematologic and gastrointestinal toxicity that is typically of a low grade as the main adverse events. To date, no biomarker has been identified which accurately predicts benefit from CDK4/6i [9]. Three CDK4/6i are currently approved universally: palbociclib, ribociclib, and abemaciclib, while dapiciclib has been approved for use in China [4].

Optimal treatment selection after progression on CDK 4/6i and endocrine therapy is less clear. The available treatment options offer limited efficacy, with significantly shorter PFS and OS [10]. There is no favored choice, especially among patients without targetable alterations (e.g., PIK3CA, AKT, or PTEN) [10,11]. Possible strategies include switching to a different class of endocrine therapy, initiating chemotherapy, or utilizing newer targeted agents [11]. Using selective estrogen receptor downregulators (SERDs) like fulvestrant require parenteral administration, and monotherapy efficacy is limited, with a median PFS of 2–3 months after CDK4/6i [12,13]. Oral SERDs seem to have greater efficacy in specific populations (e.g., mutated ESR1) and, thus far, have had limited single-agent efficacy [14]. Antibody drug conjugates (ADCs) are effective, but require intravenous infusion, and the optimal line of therapy for their use and sequencing remains unclear [15,16,17,18]. ADCs also appear to have toxicity not dissimilar to chemotherapy [19]. Drugs targeting alterations such as PI3K/AKT/mTOR inhibitors, while oral, have substantial adverse events [20], such as rash, hyperglycemia, and diarrhea, leading to significantly higher discontinuation rates due to adverse effects than the control arm [21,22].

Considering the efficacy and good tolerability of CDK4/6i, continuing their use beyond progression, either as a rechallenge after drug holiday or by changing the partner agent, is an area of interest with clear pre-clinical rationale both in breast cancer and other solid cancers [23,24]. Retrospective studies demonstrated a proof of concept of sequential use of CDK4/6i [25,26,27]. Kalinsky et al. showed that administering ribociclib + changed endocrine therapy after progression on first-line CDK4/6i and endocrine therapy was found to have a PFS benefit [28]. However, in the PACE trial, continuing palbociclib plus changing endocrine therapy to fulvestrant failed to improve PFS [29]. These studies are limited by small numbers and mixed results. Therefore, there is lack of consensus about how to best use a CDK4/6i beyond progression.

To answer the question of continued CDK4/6i use after progression, we designed this systematic review and meta-analysis. The aim of our study was to evaluate the efficacy of CDK4/6i plus a change in endocrine therapy, after progression on initial CDK4/6i + endocrine therapy in patients of ER+ HER2- advanced breast cancer and to identify patient characteristics associated with differential benefits.

## 2. Methods

The review and meta-analysis were conducted in accordance with preferred reporting items for systematic reviews and meta-analyses (PRISMA) guidelines [30].

1. Literature search and study selection

Two reviewers (NP and SK) searched MEDLINE independently to identify eligible studies. The eligibility criteria included retrospective or prospective observational studies, phase II/III randomized controlled trials, and single-arm prospective trials, in which the tested strategy was switching or continuing CDK 4/6i after progression on an initial CDK 4/6i in patients of ER+ advanced breast cancer, and which reported efficacy outcomes (overall response rate [ORR], clinical benefit rate [CBR], PFS or OS). We excluded phase I dose escalation studies even if they allowed prior CDK4/6i as they would not report the efficacy outcomes of interest.

The search was conducted from inception to August 2024. The keywords used for searching were “breast neoplasms”, “advanced breast cancer”, “metastatic breast cancer”, “palbociclib”, “ribociclib”, and “abemaciclib”, combined with Boolean operators. An additional search was performed using the websites of the American Society of Clinical Oncology (ASCO), the European Society for Medical Oncology (ESMO), and the San Antonio Breast Cancer Symposium (SABCS) to identify relevant abstracts of unpublished studies from 2019 to 2024. Citation lists of relevant systematic reviews and selected papers were also screened to increase the sensitivity of the search strategy. Any discrepancies were resolved through consensus with a third author (AM).

2. Data extraction

Two authors (NP and SK) independently obtained the required data from the included studies. Any discrepancies were resolved by consensus with a third author (AM). We extracted the following information: first author and year of publication, study design, sample size, and study-level patient characteristics, such as age of participants, presence of visceral metastases, median lines of prior treatment and median follow up, type of prior CDK 4/6i and subsequent CDK 4/6i administered, duration of CDK 4/6i exposure in prior setting, prior chemotherapy, and partner endocrine therapy (aromatase inhibitor/fulvestrant), as well as primary and secondary efficacy end points, including ORR, CBR, PFS, and OS.

3. Data synthesis and statistical analysis

Individual study characteristics were summarized using descriptive statistics. The pooled mean ORR, CBR, and PFS were calculated as the mean weighted by study sample size. Meta-regression comprising linear regression weighted by sample size (mixed effects) [31] was performed to explore the association between patient, disease and treatment-related factors, and the outcomes of interest. In light of the expectation of low statistical power, the data were interpreted quantitatively irrespective of statistical significance using thresholds described by Burnand et al. (ß coefficient ≥ 0.28 considered quantitatively significant) [32]. These analyses were performed using SPSS version 28.0 (IBM Corp, Armonk, NY, USA). Randomized studies were analyzed separately to allow the interpretation of results independently of expected bias introduced through the study design of retrospective studies. For randomized studies, we analyzed PFS as the pooled hazard ratio (HR), which was calculated by the generic inverse variance method using the random effects model. We also assessed ORR as the odds ratio (OR) using the Mantel–Haenszel method and by applying random effects modeling. Where applicable, statistically significant pooled HR and OR were defined as a two-sided *p*-value of <0.05. Statistical heterogeneity was defined as I^2^ > 50% and/or Cochran Q *p* < 0.1 [33]. Pooled analyses of randomized trials were performed using Review manager (Revman^®^) 5.4 software (Cochrane Collaboration, Copenhagen, Denmark).

## 3. Results

**Figure 1:**
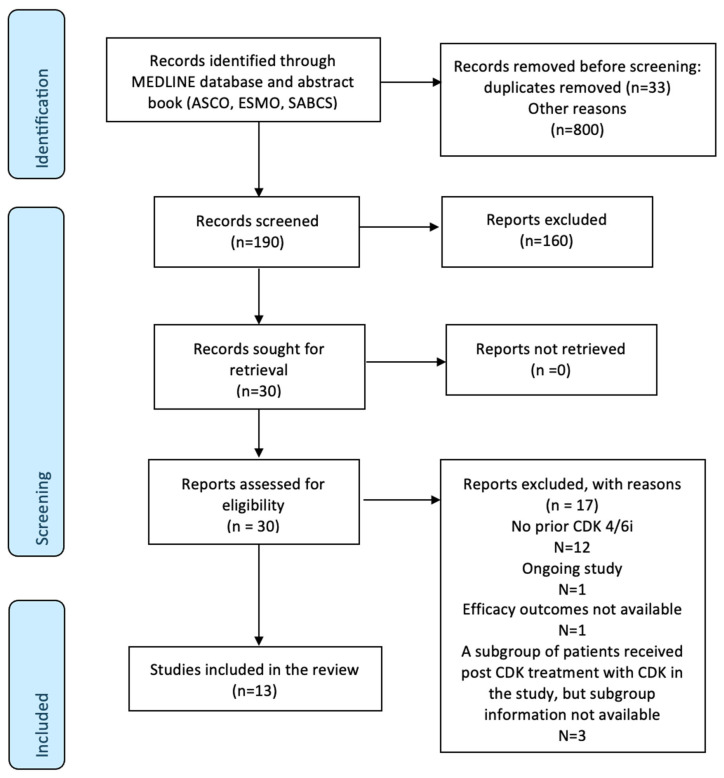
PRISMA study selection schema.

Of the 1023 studies identified initially, 13 studies with 1530 patients met the eligibility criteria. These comprised four randomized trials, two single-arm prospective studies, and seven retrospective studies [25,28,29,34,35,36,37,38,39,40,41,42,43] (see Figure 1 for study selection schema). The characteristics of the included studies are summarized in Table 1. For the PACE trial [29], the avelumab-containing arm was excluded from our analysis.

Among the included studies, the weighted median age was 58 years (interquartile range, IQR of 56–60); 50.8% (standard deviation, SD 14.16) of the patients had visceral metastases; 48% (SD 3.98) of the patients who were tested (n = 573) had ESR1 mutation. The median lines of the prior therapies were 1 (range 1–5) and almost all (96.3%, SD 5.12) received palbociclib as the first CDK4/6i. Eight studies tested a CDK4/6i switch as the intervention [25,28,35,37,39,40,41,42] (1033 patients), four continued the same CDK4/6i [29,34,36,38] with a change in the endocrine therapy backbone (497 patients), and the study by dos Anjos tested both a switch and the same CDK4/6i in two distinct cohorts [43] (see Table 1). Only three studies allowed prior chemotherapy [28,29,36]. The baseline and outcome characteristics are summarized in Table 2.

The weighted median of PFS in the CDK 4/6i intervention group was 5.3 months (IQR 3.7–6.9 months), and in the control arm, it was 5.3 months (IQR 3.6–7 months). The weighted mean (SD) ORR was 14% (SD 6.89) in the CDK 4/6i strategy group and 6% (SD 2.66) in the control group. The weighted CBRs were 35% (SD 4.68) and 28% (SD 2.02), respectively. In the subgroup of randomized controlled trials, the analysis yielded similar results, with the weighted ORR of 13.4% (SD 5.19) for the CDK4/6i-containing intervention arm and 6.47% (SD 2.66) for the control arm. Similarly, the CBR for the CDK4/6i strategy vs. control arm was 35.26% (SD 4.44) vs. CBR 28.46% (SD 2.02).

The results of the univariable meta-regression for median PFS are shown in Supplementary Table S1. We observed a quantitatively significant positive association with median age (β = 0.43) and use of non-palbociclib as an option for intervention during the switch of therapy (β = 0.364). A quantitatively significant negative association was observed with visceral metastases (β = −0.539), receipt of prior chemotherapy (β = −0.354), and the presence of mESR1(β = −0.582), palbociclib as prior CDK4/6i (β = −0.378). Multicollinearity [44] was tested for prior use of palbociclib in the initial line variable and non-use of palbociclib in the subsequent line. This yielded a variance inflation factor 2.46, which falls below the threshold for implied collinearity (VIF > 4.0 or <0.25).

The results of the meta-regression for ORR are shown in Table S2, the results of which were similar to the factors that influenced PFS.

**Figure 2:**
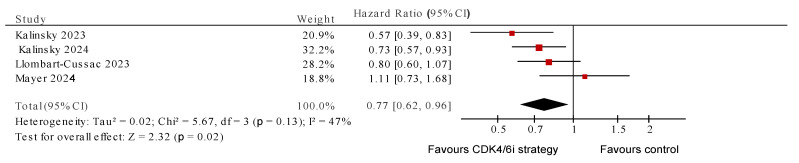
Pooled progression-free survival for randomized studies testing a CDK4/6i continuation/switch [28,29,34,35].

**Figure 3:**
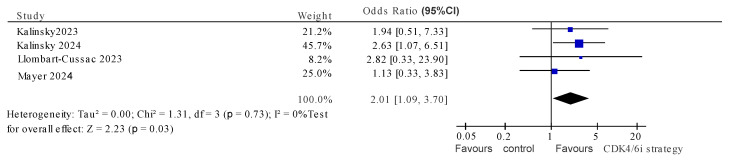
Pooled overall response rate for randomized studies testing a CDK4/6i continuation/switch [28,29,34,35].

**Figure 4:**
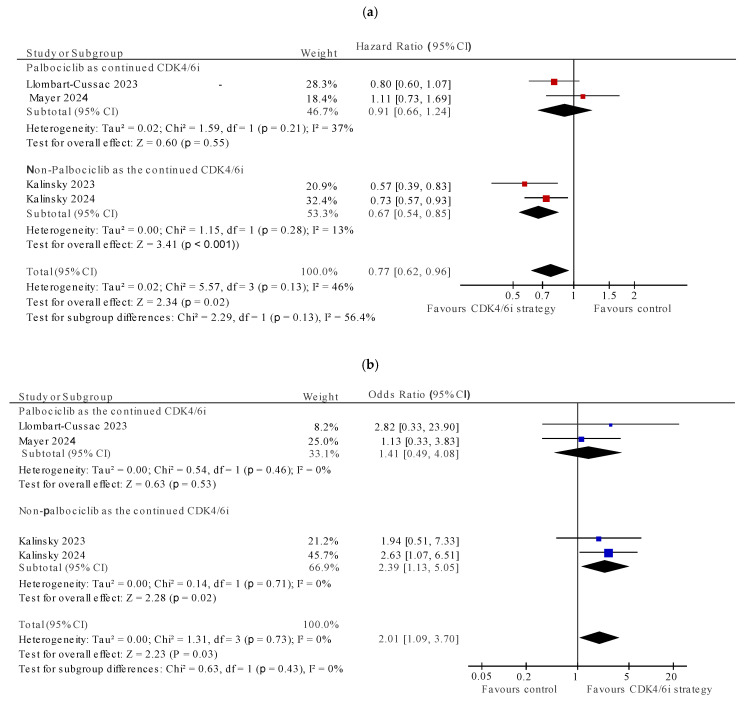
Subgroup analysis of randomized studies. (**a**) Pooled progression-free survival subgroup analysis: palbociclib as continuation strategy vs. non-palbociclib CDK4/6i; (**b**) Pooled overall response rate subgroup analysis: palbociclib as continuation strategy vs. non-palbociclib CDK4/6i [28,29,34,35].

For the four randomized studies [28,29,34,35], continuation of CDK4/6i was associated with significantly improved PFS (HR 0.77, 95% CI 0.62–0.96, *p* = 0.02) and with heterogeneity which approached, but did not meet, quantitative or statistical significance (I^2^ = 47%, *p* value for Cochran Q 0.13) (Figure 2). The pooled OR for ORR also showed positive association with continuation of CDK4/6i (OR 2.01, 95% CI 1.09–3.70, *p* = 0.03), with no evidence of heterogeneity (I^2^ = 0, Cochran Q *p* = 0.73) (Figure 3). The subgroup analysis for pooled PFS showed that using non-palbociclib as the subsequent CDK4/6i improved PFS (HR 0.67, 95%CI 0.54–0.85, I^2^ = 13%, Cochran Q *p* = 0.28), while palbociclib did not (HR 0.91, 95%CI 0.66–1.24, I^2^ = 37%, Cochran Q *p* = 0.55). Similar results were seen with the ORR subgroup analysis (Figure 4).

## 4. Discussion

The optimal second-line therapy in patients with ER+/HER2- metastatic breast cancer after progression on CDK4/6i and aromatase inhibitors remains unclear. Several approaches have been explored with one approach being the continuation of a CDK4/6i (either the same or a switch to a different agent) with a switch of endocrine therapy. This is especially relevant for those patients with no targetable alterations. However, despite several prospective and retrospective studies, there is uncertainty regarding the benefit of such an approach. In this systematic review and meta-analysis, we observed that although there was a small magnitude of improvement in response rate with this strategy compared to the control, among all the studies, there was no difference in median PFS. Acknowledging that the majority of studies included in our analysis were retrospective studies (n = 7), we performed a separate analysis of randomized controlled trials, which are considered the highest-level source of evidence [45], to allow independent interpretation of the results, thus mitigating bias and heterogeneity. Restricting the analysis to prospective randomized trials revealed modest benefits in PFS, predominantly when patients were switched from palbociclib to another CDK4/6i [28,35].

Our study identified that certain clinical characteristics can identify those who may benefit differentially from the continuation of a CDK4/6i beyond progression. Older age, low burden of disease without visceral metastasis, and no prior chemotherapy appear to be favorable characteristics for this approach. This can be explained by disease biology. Breast cancers that occur at a later age in life tend to be more indolent, driven by different epigenetic changes than younger onset breast cancers [46,47]. The absence of visceral metastases is a clinical marker known to predict a better prognosis in advanced breast cancer [48]. Fewer lines of prior therapy exposure in HR+/HER2- advanced breast cancer are associated with the development of fewer acquired resistance mechanisms [49,50]. Prior chemotherapy indicates a more aggressive biology, necessitating a quicker response to treatment; therefore, the lack of it in a prior line suggests a more indolent disease [51]. These suggest that the sequential CDK4/6i treatment studied in our review may be better suited to patients with more indolent disease. Ravani et al. [52] also found a survival benefit from continuation of a CDK 4/6i in their meta-analysis; however, unlike in this study, they did not explore the effect of clinical features such as age, sites of involvement, prior therapy, etc., which may help identify patients who would benefit more from such an approach.

We identified that mutations in ESR1 were associated with lesser benefit from continued CDK4/6i beyond progression. An ESR1 mutation is a known genomic predictor of endocrine resistance [53]. This was also seen in the study by Ravani et al. [52]. Exploratory analysis from the MAINTAIN trial suggested that the presence of ESR1 mutation in circulating tumor DNA is detrimental to ‘maintaining’ CDK4/6i treatment [28]. The recently published results of the EMBER-3 study suggest that the combination of CDK4/6i (abemaciclib) plus oral SERD (Imlunestrant) works better than CDK4/6i plus aromatase inhibitor in the second-line setting, regardless of ERS1 mutation status. However, 40% of the patients in this study did not receive CDK4/6i in the first line, making the results difficult to interpret [54]. Another strategy is to treat emergent ESR1 mutation before clinical progression with a SERD, as seen in the PADA-1 trial, which assessed palbociclib plus ET, with a switch from an aromatase inhibitor to fulvestrant when ESR1 mutation was detected in circulating tumor DNA [55]. The results of SERENA-6 (NCT04964934), which tests a similar approach, using Camizestrant, an oral SERD in emergent ESR1 mutation (instead of fulvestrant), are awaited. Changing the CDK4/6i component from palbociclib to another CDK4/6i appeared to be a better option than simply continuing palbociclib with a change in endocrine therapy alone. The majority (96%) of the patients included in our analysis received palbociclib in the first line. Furthermore, seven out of eight studies [25,26,28,35,37,39,42] evaluating a switch of CDK4/6i used palbociclib as the first-line therapy, while information on first-line CDK4/6i is missing for the eighth study [41]. This limits our ability to draw strong conclusions on the appropriate sequencing of CDK 4/6i, as almost no patients received a non-palbociclib CDK4/6i in the first line.

There was considerable inter- and intra-study heterogeneity in the endocrine therapy partner in subsequent line therapy, during the CDK4/6i continuation/switch. Most studies favored the use of fulvestrant as the partner [29,35,38,39,42], while some allowed both fulvestrant and aromatase inhibitors [25,28,34,36,37,40], and others permitted abemaciclib alone [25,37]. This may have impacted the results, as fulvestrant is known to be active in tumors resistant to aromatase inhibitors [56]. However, considering that two of the randomized controlled trials [29,34] included in this meta-analysis studied the use of palbociclib in the subsequent line, with the change in endocrine therapy, and that this strategy did not show a benefit in pooled subgroup analysis, it is likely that the benefits are more driven by the CDk4/6i component than the choice of endocrine partner.

There are known pharmacological differences among the three agents, especially the differential inhibition of CDK4 compared to CDK6 [57]. This allows for continuous administration of abemaciclib, as it does not have a dose-limiting toxicity of myelosuppression [58]. Continuous CDK4/6 inhibition results in a sustained inhibition of ER+ breast cancer cells in xenograft models, which may have clinical relevance [59,60]. Randomized controlled trials conducted for CDK4/6i in the first-line setting demonstrated an OS advantage of this approach, compared to controls, for ribociclib and abemaciclib, but not palbociclib [61,62,63]. Possible theories for this include differences in trial or statistical design, censoring, post-progression therapies, endocrine sensitivity, and/or pharmacological efficacy among CDK4/6i [64]. A recent network meta-analysis by Kappel et al. [9], however, demonstrated similar efficacy for PFS and OS for all three CDK4/6i in the first-line setting. That said, similar efficacy upfront may not be a valid predictor of efficacy after development of resistance. This has been demonstrated in in vitro models, where the pathways of resistance seen are distinct for palbociclib vs. abemaciclib resistance. Palbociclib-resistant cells continue to respond to abemaciclib as a sequential therapy, but not vice versa [65,66].

Counterintuitively, the use of palbociclib in the prior line as a variable appeared to predict for a lesser effect of subsequent CDK4/6i; this observation may be confounded by other factors even though the VIF was only moderate. Unfortunately, due to a small number of studies available for analysis, multivariable regression could not be performed due to the risk of poor model fit.

Our study supports the rationale of continued CDK4/6 inhibition beyond progression as a viable option for carefully selected patients of ER+ advanced breast cancer. Other options for second-line therapy may require a specific molecular marker, such as capivasertib or alpelisib in combination with fulvestrant in PI3KCA/PTEN/mTOR altered tumours and trastuzumab deruxtecan inHER2-low cancers. The PFS benefits of these treatment options are also relatively modest, with a 4–5-month absolute difference [22,67,68]. Moreover, some of these options have shown a significant burden of side effects and discontinuation rates due to toxicity (fulvestrant–alpelisib: 26%), (fulvestrant–capivasertib: 13% and trastuzumab deruxtecan: 14%) [22,67,68], as compared to abemaciclib + fulvestrant discontinuation rates of 6%, as seen in the postMONARCH study [35]. Everolimus plus endocrine therapy and chemotherapy are more conventional options; however, they are also limited by side effects (discontinuation rates of everolimus- based therapy: 19%) [10,69]. The results of the SONIA trial support the use of endocrine therapy alone in the first line with CDK4/6i plus fulvestrant upon progression. This adds to the importance of sequencing therapies and maximizing benefit, while minimizing side effects [70]. Future studies should focus on optimal sequencing of the available agents in this domain.

This study has limitations. Most of the included studies were retrospective with a small sample size, which affects the quality of the analysis and its inference. Multivariable regression analysis could not be performed due to the limited number of included studies. There was moderate heterogeneity seen in the PFS analysis of randomized studies, as well as potential multicollinearity with initial use of palbociclib in the meta-regression analysis. An individual participant-level data analysis would have added to the robustness of the results.

## 5. Conclusions

In summary, the systematic review and meta-analysis have shown that continuation of CDK4/6i beyond progression likely does not provide meaningful benefit in unselected patients. It is possible that those with indolent disease biology and those for whom there are no targetable alterations may derive more benefit. For such patients, a switch to a different CDK4/6i as well as a change in endocrine therapy can be considered.

## Figures and Tables

**Table 1:** Characteristics of included studies.

	Study ID	Study Design	Sample Size and Patient Population	Intervention Arm	Control Arm	Primary Endpoint
1.	Mayer 2024 [29] * (PACE)	Phase II RCT	Ni = 111 Nc = 55 ER+ HER2- aBC, PD on AI + CDK4/6i (at least 6 m of therapy in the metastatic setting or ≤12 m of CDK4/6i as adjuvant); 1 CT and ≤2 ET allowed	Palbociclib + fulvestrant	Fulvestrant	PFS
2.	Kalinsky 2023 [28] (MAINTAIN)	Phase II RCT	Ni = 60 Nc = 59 ER+ HER2- aBC, PD on ET + CDK4/6i, ≤1 CT allowed	Ribociclib + fulvestrant/exemestane	Placebo + fulvestrant/exemestane	PFS
3.	Kalinsky 2024 [35] (postMONARCH)	Phase III RCT	Ni = 182 Nc = 186 ER+ HER2- aBC, PD on CDK4/6i + AI as 1st-line therapy or relapse on/after a CDK4/6i + ET as adjuvant therapy for eBC. No other prior treatment for aBC was permitted.	Abemaciclib + fulvestrant	Placebo + fulvestrant	PFS
4.	Llombart-Cussac 2023 [34] (PALMIRA)	Phase II RCT	Ni = 136 Nc = 62 women with ER+ HER2- aBC who had PD on 1st-line palbociclib plus ET with benefit (SD or better response for ≥24 weeks) or in eBC adjuvant P + ET for ≥12 m, and within 12 m of completion	Palbociclib + 2nd-line ET	2nd-line ET alone	PFS
5.	Albanell 2023 [36] (BioPER)	Phase II single arm	N = 33 Women with ER+ HER2- aBC, PD on palbociclib + ET with documented benefit (SD or better response for ≥24 weeks), intervening treatment not allowed	Palbociclib + ET of physician’s choice	-	CBR and % of tumors with baseline loss of Rb protein expression
6.	Tao 2022 [38]	Phase II single arm	N = 58 ER+ HER2- aBC, PD on palbociclib + AI (after 1st-line palbociclib + AI at least for ≥24 weeks)	Palbociclib + fulvestrant	-	PFS
7.	Mariotti 2019 [37]	Retrospective	N = 19 ER+ HER2- aBC, PD on palbociclib + ET	Abemaciclib +/− ET	-	-
8.	Wander 2021 [25]	Retrospective	N = 87 ER+ HER2- aBC, PD on palbociclib/ribociclib plus ET 71.3% received some intervening therapy	Abemaciclib +/− ET	-	-
9.	Eziokwu 2021 [40]	Retrospective	N = 30 women with ER+ HER2- aBC, PD on CDK4/6i where CDK4/6i was continued beyond PD, intervening treatment not allowed	CDK4/6i + ET ^b^	-	PFS
10.	Tamragouri 2019 [42]	Retrospective	N = 21 ER+ HER2- aBC, PD on palbociclib + ET	Abemaciclib +/− fulvestrant	-	-
11.	dos Anjos 2019 [43], cohorts 2 and 3	Retrospective	N = 43 in cohort 2 N = 84 in cohort 3 ER+ HER2- aBC, PD on CDK4/6i + ET	Cohort 2: same CDK4/6i + change in ET Cohort 3: different CDK4/6i +/− ET	-	Time to subsequent therapy TTST
12.	Seki 2022 [39]	Retrospective	N = 25 ER+ HER2- aBC, PD on palbociclib + ET	Abemaciclib +/− fulvestrant	-	-
13.	Kruse 2023 [41]	Retrospective	N (cohort 1): 165 N (cohort 2): 115 ER+, HER2-, and documentation of an abemaciclib- containing regimen and at least 1 other anti-cancer systemic therapy	Cohort 1: subsequent CDK4/6i after 1st-line CDK4/6i Cohort 2: subsequent CDK4/6i after 2nd-line CDK4/6i	-	PFS

Abbreviations: aBC, advanced breast cancer; AI, aromatase inhibitor; CBR, clinical benefit rate; CDK4/6i cyclin-dependent kinase 4/6 inhibitor; eBC, early breast cancer; ER+, estrogen/progesterone receptor-positive; ET, endocrine therapy; HER2-, Human Epidermal Growth Receptor 2-negative; N, sample size; Ni, sample size in interventional arm; Nc, sample size in control arm; PD, progression of disease; PFS, progression-free survival; RCT, randomized controlled trial. * The third arm of the study with palbociclib + avelumab was excluded from analysis. ^b^ 94% (28 out of 30) patients received palbociclib in subsequent therapy; 6% (2 out of 30) received abemaciclib.

**Table 2:** Results of included studies.

Study ID	Age (Median Years)	Visceral Disease	mESR1	Prior Palbociclib	Prior CT	CDK 4/6i ≥ 12 Months	PFS (Months); HR, (CI)	OS, Median (Months); HR (95% CI)	ORR (%)	CBR (%)
Mayer 2024 * [29] (PACE)	57	59.6%	54%	92.8%	16.3%	77.7%	4.6 vs. 4.8; HR, 1.11 (0.79 to 1.55)	24.6 vs. 27.5; HR 1.02 (0.67 to 1.56)	9% vs. 7% ^a^	32.4% vs. 29.1 ^a^ %
Kalinsky 2023 [28] (MAINTAIN)	57	59.7%	42.3%	86.6%	9.2%	67%	5.3 vs. 2.8; HR, 0.57 (0.39–0.85	-	20% vs. 11% *p* 0.51	43% vs. 25% *p* 0.06
Kalinsky 2024 [35] (postMONARCH)	61	60.5%	46.8%	59%	0	76.1%	6 vs. 5.3; HR, 0.73 (0.57–0.95)	immature	17% vs. 7% *p* 0.01	-
Lombart-Cussac 2023 [34] (PALMIRA)	59	61.1%	-	100%	0	85.9%	4.2 vs. 3.6; HR, 0.8 (0.6–1.1)	28.3 vs. 28.8; HR, 1.06 (0.75–1.51)	6.4% vs. 2.3%	33% vs. 29.5%
Albanell 2023 [36] (BioPER)	59.5	78.1%	52%	100%	12.5%	-	2.6 (95%CI 1.8–6.7)	23.9 (95% CI 16.4-NE)	6.3%	34.4%
Tao 2022 [38]	57.9	-	-	-	-	-	3.7	-	-	-
Mariotti 2019 [37]	57	73.8%	-	100%	-	-	7.0 (95%CI 1.8–12.1)	-	0	33%
Wander 2021 [25]	52	-	-	100%	-	-	5.3 (95% CI 3.5–7.8)	17.2 (13.2-NR)	-	-
Eziokwu 2021 [40]	47.5	43.3%	-	100%	-	-	11.8 (95%CI 5.34–13.13)	45.4	-	-
Tamragouri 2019 [42]	57.8	-	-	100%	-	-	-	-	-	29%
dos Anjos 2019 [43] Cohort 2	-	-	-	-	-	-	4.5 (95%CI 3.3–7.6)	-	-	-
dos Anjos 2019 [43] Cohort 3	-	-	-	-	-	-	4.4 (95%CI 3.8–5.9)	-	29%	-
Seki 2022 [39]	69	68%	-	-	-	-	5.3 (95%CI 3.08–7.52)	-	16%	44%
Kruse 2023 [41]	cohort 1: 63.3 cohort 2: 66.5	-	-	-	-	-	cohort 1: 12.7 (95%Ci 9.4, 20) cohort 2: 9.1 (95%CI 5.2, 12.1)	-	-	-

Abbreviations: CDK4/6i, cyclin-dependent kinase 4/6 inhibitor; CBR, clinical benefit rate; CI, confidence interval; CT, chemotherapy; mESR1, mutated Estrogen Receptor 1; HR, hazard ratio; ORR, overall response rate; OS, overall survival. * 90% confidence intervals (CI) in the study; remaining studies have 95% CI; ^a^ *p* value not available.

## Data Availability

As this is a systematic review and meta-analysis, the data collected already exist in the public domain in the form of published studies, which can be obtained from the references cited in the manuscript or by contacting the corresponding authors of the concerned studies directly.

## Supplementary Materials

The following supporting information can be downloaded at: https://www.mdpi.com/article/10.3390/cancers17101609/s1. Supplementary Table S1: Meta regression for median progression free survival; Supplementary Table S2: Meta regression for mean overall response rates.
