# Supplementary material for: Continuing Cyclin-Dependent Kinase 4/6 Inhibitors Beyond Progression in Advanced Breast Cancer: A Meta-Analysis
Source: Cancers (Basel). 2025 May 9;17(10):1609. doi: 10.3390/cancers17101609 (PMC12109698; doi:10.3390/cancers17101609)
Supplement: Supplementary file 1 [file cancers-17-01609-s001.zip › cancers-3597414-supplementary.pdf]

Variable	Beta coefficient (β)	p value
Median age	0.43	0.207
Visceral metastases	-0.539	0.168
Median lines of treatment	-0.073	0.927
Prior line palbociclib (%)	-0.378	0.136
Prior CDK 4/6i duration	-0.226	0.626
CDK4/6i tested, by proportion of those who did not receive palbociclib	0.364	0.301
Prior chemotherapy	-0.354	0.769
mESR1 (% of patients)	-0.582	0.418

Supplementary table 1: Meta regression for median progression free survival

Variable	Beta coefficient (β)	p value
Median age	-0.073	0.877
Visceral metastases	-0.645	0.118
Median lines of prior treatment	-0.068	0.957
Prior palbociclib (%)	-0.828	0.042
Duration of prior CDK 4/6i	0.174	0.742
CDK4/6i tested, by proportion of those who did not receive palbociclib	0.794	0.019
Prior chemotherapy	-0.880	0.315
mESR1 (% of patients)	-0.958	0.042

Supplementary table 2: Meta regression for mean overall response rates
